# Trends in silicosis prevalence and the healthy worker effect among gold miners in South Africa: a prevalence study with follow up of employment status

**DOI:** 10.1186/s12889-015-2566-8

**Published:** 2015-12-18

**Authors:** David Knight, Rodney Ehrlich, Katherine Fielding, Hannah Jeffery, Alison Grant, Gavin Churchyard

**Affiliations:** Centre for Environmental and Occupational Health Research, School of Public Health and Family Medicine, University of Cape Town, Cape Town, South Africa; International SOS, Singapore, Singapore; London School of Hygiene and Tropical Medicine, London, United Kingdom; The Aurum Institute, Johannesburg, South Africa

**Keywords:** Healthy worker survivor effect, Mining, Pneumoconiosis, Thibela TB, Tuberculosis, Respirable crystalline silica, Silica

## Abstract

**Background:**

Given the intimate association between silicosis and tuberculosis, understanding the epidemiology of the South African gold mining industry silicosis epidemic is essential to current initiatives to control both silicosis and tuberculosis in this population, one of the most heavily affected globally. The study’s objectives were to compare the prevalence of silicosis among working black gold miners in South Africa during 2004–2009 to that of previous studies, including autopsy series, and to analyse the influence of silicosis and/or tuberculosis on exiting employment.

**Methods:**

Routine chest radiographs from a cohort of gold miners were read for silicosis by an experienced reader (I), and a subset re-read by a B-trained reader (II). Two methods of presenting the readings were used. Additionally, with baseline status of silicosis and previous or active tuberculosis as predictors, survival analysis examined the probability of exiting the workforce for any reason during 2006–2011.

**Results:**

Reader I read 11 557 chest radiographs and reader II re-read 841. Overall, silicosis prevalence (ILO ≥1/0: 5.7 and 6.2 % depending on reader method) was similar to the age adjusted prevalence found in a large study in 1984 (5.0 %). When comparison was restricted to a single mine shaft previously studied in 2000, a decline in prevalence (ILO ≥1/1) was suggested for one of the reading methods (duration adjusted 20.5 % vs. 13.0 % in the current study). These findings are discordant with a long-term rising autopsy prevalence of silicosis over this period. Overall, relative to miners with neither disease, the adjusted hazard ratio for exiting employment during the follow-up period was 1.54 for baseline silicosis [95 % confidence interval (CI) 1.17, 2.04], 1.71 for tuberculosis (95 % CI 1.51, 1.94) and 1.53 for combined disease (95 % CI 1.20, 1.96).

**Conclusions:**

This study found, a) there was no significant decline in overall silicosis prevalence among working black miners in the South African gold mining industry between 1984 and 2004–2009, and b) a possible decline at one mine shaft more recently. In the absence of evidence of declining respirable silica concentrations between the 1980s and 2000s, the trends found are plausibly due to a healthy worker survivor effect, which may be accelerating.

**Electronic supplementary material:**

The online version of this article (doi:10.1186/s12889-015-2566-8) contains supplementary material, which is available to authorized users.

## Background

In 2003 the South African mining industry committed itself to eliminating silicosis, the target being no new cases by 2013 among miners unexposed to silica dust prior to 2008 [1]. In 2012 the Ministers of Health of the Southern African Development Community (SADC) signed a declaration committing their countries to reducing the burden of mining related lung disease [2]. Included among the mechanisms for achieving this reduction was improved surveillance of silicosis and tuberculosis, two diseases linked through their association with silica dust exposure. An increased risk of tuberculosis has been shown in silica exposed populations both with and without radiological silicosis, [3–5] an effect aggravated in the Southern African mining population by an HIV infection prevalence as high as 27 % [6]. HIV infection is not only a strong independent risk factor for tuberculosis, but when combined with silicosis interacts multiplicatively to produce a very high relative risk of tuberculosis compared to miners with neither HIV infection nor silicosis [7].

Measurement of trends in silicosis prevalence is therefore of pressing relevance in the South African gold mining industry, the main source of silicosis in the region and an amplifier of tuberculosis risk. Such information is needed, for example, to inform the plan of the Global Fund to Fight HIV, Tuberculosis and Malaria to spend ZAR 500 (US$36) million on the screening, and where needed treatment, of 500 000 miners, ex-miners and peri-mining communities for tuberculosis [8].

Silicosis surveys of working miners are scarce. A large 1984 workforce survey of black miners^1^ at a gold mine in the Free State province [9, 10] found a minimum crude silicosis prevalence of 1.36 %, based on a radiological profusion grade ≥1/0 (R. Cowie, personal communication, 2014) on the International Labour Organisation (ILO) scale [11]. Silicosis prevalence rose with age, reaching 15 % in miners aged 51 to 55 years [10]. A smaller survey in 2000 at a different gold mine of 520 black miners, [12, 13] aged at least 37 years and with mean duration of service of 21.8 years, found a high prevalence of silicosis (ILO ≥1/1) of 18.3 to 19.9 % (depending on the reader).

These latter findings are consistent with studies conducted in the 1990s and early 2000s of *former* gold miners, from the Transkei region of South Africa, [14] Botswana, [15] and Lesotho, [16, 17] in which silicosis prevalence ranged from 22 to 36 % depending on the study and reader. The findings are also consistent with a rising proportion of silicosis found at autopsy in (mainly in-service) black gold miners [18].

A study of community-wide tuberculosis preventive therapy conducted between 2006 and 2011 provided an opportunity to update estimates of silicosis prevalence among working black miners [19, 20].

The objective of the analysis presented in this report was therefore to measure the prevalence of silicosis in a representative multi-company sample of working black gold miners, and to compare the prevalence with that found in previous studies. In the course of the study a second objective was added, namely, to analyse the influence of baseline status of silicosis and/or active or previous tuberculosis on the likelihood of exiting employment during the approximately 3 year follow-up period of the tuberculosis prevention study.

## Methods

The Thibela TB (“Stop TB”) study [19, 20] was a cluster-randomised trial conducted in 20 shafts (15 “clusters”) operated by three large gold mining companies in three mining regions of South Africa. The aim of that study was to test tuberculosis prophylaxis with isoniazid at the community (mine) level. A cluster was defined as one or more mine shafts with an associated single hostel. For inclusion in the study, the shaft must have been operating for more than 5 years and have had a minimum of 1000 miners.

As part of the Thibela TB study, a baseline survey using a random sample of approximately 1000 miners in each cluster (a subset of the total workforce) was conducted, staggered over calendar time (2006–2009). Enrolment was a two-stage process whereby a) miners randomly selected from the workforce were invited to attend the study centre post-shift, and b) those who attended were invited to participate in the study and were enrolled after giving informed consent. Information on demographic and other risk factors associated with tuberculosis was obtained along with the most recent routine chest radiograph taken by the mine medical service as part of the regular screening of miners for occupational lung disease. Radiographs were dated between June 2004 and February 2009, with a median interval of 5.3 months before the baseline interview (maximum interval 21 months). HIV results were not available for most of the participants.

Radiographs were read for silicosis according to the ILO Classification [11] and for signs of tuberculosis using a schema designed for the Thibela TB study. This schema classified specific abnormalities as indicative of either “active” or “previous” tuberculosis. All radiographs were read by a nursing health professional (reader I) with 30 years of experience in reading radiographs for tuberculosis and silicosis. For the purpose of the silicosis study an experienced occupational medicine physician and trained B-reader (reader II – author RE) read a) all radiographs reported as silicosis ILO grade ≥0/1 by reader I, and b) a random sample of those reported as completely normal by reader I (25 per cluster). Almost all radiographs were read as digital images.

### Analysis

For comparability with previous studies of in-service workers, analyses were restricted to male, black employees who reported having ever worked underground. “Contractors”, i.e. workers employed by contracting companies or labour brokers rather than directly by the mining company, were excluded. Silicosis was defined as either ILO ≥1/0 or ILO ≥1/1, also for comparability with previous studies. For the subset of radiographs read by both readers inter-reader agreement was assessed by sensitivity and specificity (against reader II), percentage agreement and the kappa statistic. For reader I, the prevalence of silicosis and associated 95 % confidence interval (CI) was calculated, stratified by age group and number of years since first employed, and adjusted for clustering using robust standard errors.

A “combined” reading prevalence was then calculated in the following way: a) since reader II re-read all of reader I’s *positive* films (as defined above), reader I’s gradings of these films were replaced by reader II’s gradings where they differed; b) since reader II read only a fraction of reader I’s *negative* films, the proportion of these films read as positive by reader II was added to the prevalence calculated under a) to yield a “combined reading” prevalence. Because of the sampling method used, 95 % confidence intervals were not calculated for this combined prevalence.

Cox proportional hazards regression, with robust standard errors to account for clustering, was used to assess whether baseline silicosis (ILO ≥1/1) and /or tuberculosis (using a sensitive definition of any radiographic signs or self-report, and based on reader I’s readings) was associated with time to exiting the workforce for any reason.

Time at risk was measured from the date of the baseline survey (the first cluster survey starting in 2006) to the earliest date of leaving the workforce or to the end of the cluster follow-up in the Thibela TB study, approximately 3 years after enrolment. Date of leaving the workforce was obtained from monthly Human Resources records, with reason for leaving being unavailable for some of the mines. An adjusted analysis was performed, controlled for age, number of years since first employed, country of origin, frequency of underground work (full time versus part time underground) and occupational level (unskilled, skilled or professional).

## Results

### Response

**Fig. 1:**
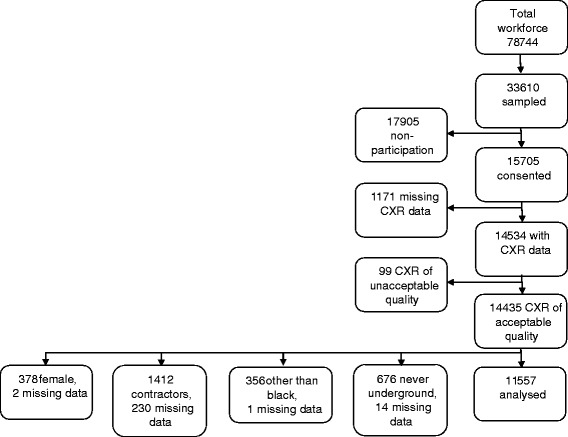
Baseline survey sampling scheme

The total sample recruited into the Thibela TB trial, the subset included in the silicosis analysis, response rates and exclusions are detailed in Fig. 1. The radiographs of 11,557 miners were included in this analysis. Because of missing data on covariates, the sample size varied slightly in different analyses.

### Inter-reader agreement

A total of 841 radiographs from the above sample of miners were read for silicosis by both readers (Additional file 1: Table S1a). Taking reader II as the “gold standard” and the ILO threshold for silicosis as  ≥1/1, reader I achieved 92 % sensitivity; i.e. if reader II read the radiograph as positive, reader I almost always read it as positive as well. By contrast, reader I achieved only 58 % specificity; i.e. if reader II read the radiograph as negative, reader I agreed in somewhat over half the cases. As a result, agreement between readers was only 67 %, with a kappa value of 0.36. Agreement deteriorated appreciably when reader I identified any signs of pulmonary tuberculosis as present on the radiograph (Additional file 1: Table S1b). In particular, reader I’s specificity for silicosis (as defined above) dropped to 21 % when tuberculosis was reported by reader I as present, compared to 65 % when it was reported as absent. Sensitivity for silicosis remained high.

### Prevalence

Table 1 details the radiographic prevalence of silicosis by age and years since first employment in the mining industry for both reading sets. At ILO grade ≥1/1 the overall prevalence of silicosis was 4.1 % by reader I and 5.7 % by the combined method. Silicosis was uncommon among participants under 45 years of age or with fewer than 20 years since starting in the industry, increasing sharply thereafter.

**Table 1:** Prevalence of radiological silicosis by age category and years since first employment in the mining industry, both reading sets

		Reader I				Combined reading			
		ILO grade ≥1/0		ILO grade ≥1/1		ILO grade ≥1/0		ILO grade ≥1/1	
	N	n	Prevalence % (95 % CI)^a^	n	Prevalence % (95 % CI)^a^	n	Prevalence %	n	Prevalence %
Total	11,557	659		478					
By age (years)									
18–44	6468	128	2.0 (1.5, 2.7)	85	1.3 (0.9, 1.8)	56	0.9	98	1.5
45+	4994	526	10.5 (8.6, 12.9)	389	7.8 (6.1, 10.0)	659	13.2	551	11.0
Total^b^	11,462	654	5.7 (4.6, 7.1)	474	4.1 (3.2, 5.3)	715	6.2	649	5.7
By duration since first employment (years)									
≤20	5456	81	1.5 (1.1, 2.1)	52	1.0 (0.7, 14.0)	27	0.5	48	0.9
21+	6020	573	9.5 (7.8, 11.7)	423	7.0 (5.5, 9.0)	681	11.3	618	10.3
Total^c^	11,476	654	5.7 (4.6, 7.1)	475	4.1 (3.2, 5.3)	708	6.2	666	5.8

*CI* confidence interval. All figures rounded to one decimal

^a^95 % confidence intervals adjusted for clustering using robust standard errors

^b^Restricted to those with age known (age unknown for *n* = 95)

^c^Restricted to those with duration in workforce known (years since first employment unknown for *n* = 81)

### Comparison with previous studies of active gold miners

The crude silicosis prevalence in the 1984 study (1.4 %), using ILO ≥1/0 as the definition, was low due to the very high proportion of young workers (Table 2) [9, 10]. Once age was standardised to the current study, the 1984 silicosis prevalence was 5.0 %, lower than the ILO ≥1/0 prevalences in the current study of 5.7 and 6.2 % (depending on reading method). Based on the confidence interval around Reader I’s prevalence of 4.6 to 7.1 % (Table 1), the difference was not statistically significant. (As mentioned above, reader II’s confidence interval could not be calculated).

**Table 2:** Comparison of prevalence of silicosis by age: 1984 study [7, 8] versus current study (ILO grade ≥1/0)

1984 study				Current study				
Age (years)	N (%)	Cases n	Silicosis prevalence % (95 % CI)^a^	Age (years)	N	Age distribution %	Silicosis prevalence (reader I) % (95 % CI)^b^	Silicosis prevalence (combined reading) (%)
≤45	125,038 (94.2)	1009	0.8 (0.8, 0.9)	≤44	6468	56.4	2.0 (1.5, 2.7)	0.9
46+	7726 (5.8)	801	10.4 (9.7, 11.1)	45+	4994	43.6	10.5 (8.6, 12.9)	13.2
Crude	132,765	1810	1.4 (1.3, 1.4)	Crude	11,462	100	5.7 (4.6, 7.1)	6.2
Adjusted^c^			5.0					

All figures rounded to one decimal

^a^95 % confidence intervals using binomial exact standard errors

^b^95 % confidence intervals adjusted for clustering using robust standard errors

^c^Age standardised to current study age distribution

For comparison with the 2000 study (*n* = 510), [12, 13] analysis of the current readings was restricted to the same shaft and approximately the same age group as in the earlier study (*n* = 230) and a silicosis definition of ≥1/1 (Table 3). Prevalence in the current study was stratified by time since starting in the industry whereas duration of service was used in the earlier study. The adjusted prevalence in the 2000 study, using the Thibela distribution of duration, was 20.5 %. This was higher than the prevalence in this study based on reader I’s readings (18.7 %) but fell within the 95 % confidence interval of the latter prevalence (14.2–24.2 %). By contrast, the prevalence based on the combined reading was substantially lower than that in the 2000 study (13 % vs. 20.5 %).

**Table 3:** Comparison of prevalence of silicosis by duration: 2000 study [12, 13] versus current study (ILO grade ≥1/1)

2000 study (≥37 years of age)				Current study (≥40 years of age)^a^			
Duration years^b^	N %	Cases	Silicosis prevalence % (95 % CI)^c^	Duration years^b^	N %	Silicosis prevalence (reader I) % (n-cases) (95 % CI)^c^	Silicosis prevalence (combined reading) %
≤20	179	14	7.8 (4.3, 12.8)	≤20	48	12.5 (6)(4.7, 25.2)	8.3
21+	331	79	23.9 (19.4, 28.8)	21+	182	20.3 (37)(14.7, 26.9)	14.4
Crude	510^d^	93	18.2 (15.0, 21.9)	Overall	230	18.7 (43)(13.9, 24.3)	13.0
Adjusted^e^			20.5				

*CI* confidence interval. All figures rounded to one decimal

^a^For comparability, restricted to participants in this study from the same shaft and ≥40 years of age (*N* = 230)

^b^Duration of exposure (2000 study), duration since first employment (current study)

^c^95 % confidence intervals using binomial exact standard errors

^d^Numbers used to derive Fig. 1 in 2000 study obtained from authors

^e^Standardised to duration distribution of current study and the same shaft

### Exit from workforce

Survival analysis, with exit from the workforce for any reason as the outcome, was carried out to test the hypothesis that a healthy worker survivor effect was one of the explanations of the declining silicosis prevalence. Of 11 529 miners contributing 25 691 person-years at risk, 2647 left the workforce over a median follow-up period of 2.4 years (interquartile range 2.03–2.71 years).

The adjusted analysis is presented in Table 4. After adjustment for age, years since first employed, country of origin, time spent underground and occupational level, miners with silicosis alone (≥1/1 and above) at baseline were 1.54 times more likely to exit employment than those with neither silicosis nor tuberculosis. Similarly, miners classified with active or previous tuberculosis at baseline (whether on radiograph or history) without silicosis were 1.71 times more likely to exit employment than those with neither tuberculosis nor silicosis. The hazard ratio for those with evidence of both silicosis and tuberculosis at baseline was 1.53; *p*-value for interaction <0.001. This is discussed further below.

**Table 4:** Survival analysis (Cox regression) of time to exiting the workforce (for any reason) during follow up (*N* = 11 529)^a^

Model	Disease	Presence	Number	Left workforce	Cumulative proportion leaving workforce by 2 years	HR (95 % CI)	*P* value (Wald)	Adjusted HR (95 % CI)	*P* value (Wald)
1	Silicosis (grade 1/1+)	No	11,053	2473	0.175	1.0	0.001	1.0	0.12
		Yes	476	174	0.252	1.69 (1.34, 2.11)^b^		1.18 (0.96, 1.47)^c^	
	TB^d^	No	8512	1623	0.150	1.0	<0.001	1.0	<0.001
		Yes	3017	1024	0.260	1.97 (1.77, 2.19)^b^		1.64 (1.47, 1.84)^c^	
	Interaction between silicosis and active/previous TB								
2	Silicosis (grade 1/1+) and TB^d^	No silicosis, no TB	8230	1525	0.147	1.0	<0.001	1.0	<0.001
		Silicosis, no TB	282	98	0.233	1.97 (1.51, 2.57)^b^		1.54 (1.17, 2.04)^e^	
		No silicosis, TB	2823	948	0.258	2.01 (1.79, 2.26)^b^		1.71 (1.51, 1.94)^e^	
		Silicosis plus TB*	194	76	0.281	2.26 (1.84, 2.78)^b^		1.54 (1.20, 1.96)^e^	

*HR* hazard ratio, *CI* confidence interval, *TB* tuberculosis

^a^Based on reader I’s readings

^b^Adjusted for clustering using robust standard errors

^c^Adjusted for age category, years since first employed, country of origin, time spent underground, occupational level, silicosis/TB (active or previous) as appropriate, and clustering using robust standard errors. (*n* = 11 371)

^d^TB defined as either previous or active TB at baseline by medical history or CXR by reader I

^e^Adjusted for age category, years since first employed, country of origin, time spent underground, occupational level, and clustering using robust standard errors. (*n* = 11 371)

*p-value for interaction between active/previous TB and silicosis <0.001 adjusting for clustering only, and *p*-value <0.001 from the fully adjusted model

## Discussion

### Comparison with 1984 study

This study of over 11 500 black miners in three large South African gold mining companies showed no decline in silicosis prevalence from that recorded in one large company in 1984. Age adjusted prevalence was actually higher in this study, although not statistically so. The 1984 study relied on readings of 100 mm × 100 mm miniature film radiographs, which under research conditions have been shown to have high sensitivity for full size films [9, 10], and relatively high sensitivity for autopsy detected silicosis [21]. However, the 1984 prevalence might have been an underestimate if cases were missed by the use of miniature radiographs.

### Comparison with 2000 study

In the comparison with a more recent study of working miners [12, 13], a difference was evident between the prevalence found in that study and the prevalence estimated from the combined reading in this study (20.5 % vs. 13 %). No significant difference was apparent using reader I’s findings. The time elapsed between the two studies was 4 to 8 years, the shaft was the same, the images were full size, the same age stratum was compared and reader II had read in both studies. One difference was that the current study recorded time since first employment rather than total duration of employment, such that the current study might have overestimated employment duration for miners with interrupted service. Given that duration was a binary variable, this is unlikely to have resulted in substantial misclassification.

### Discordance with other sources of information on silicosis prevalence

However, caution has to be exercised in generalising about a recent decline in silicosis prevalence at one shaft to the industry as a whole. Besides the discrepancy between readers, the sample of workers from that shaft available in the current study was small and the manner used to compute the combined prevalence did not allow for statistical significance testing of the difference. Further, the silicosis prevalences found in individual clusters in this study, as read by reader 1 and unadjusted for age or duration, varied six-fold from lowest to highest (not shown in this report).

Nonetheless, the possibility of a recent decline at one shaft and the flat trend overall compared to the 1984 study are discordant with the rising proportion of autopsy detected silicosis in black miners [18]. South Africa has long had a statutory autopsy system for miners and ex-miners [22]. Autopsies are performed irrespective of the cause of death, and in black miners most are carried out on miners who die during their period of employment. In the latest analysis of the autopsy database [18], natural deaths and particularly HIV related and TB deaths were excluded, leaving effectively a “random” sample of employed miners assuming that risk of a non-natural mining death is not correlated with silicosis risk. Reading off Fig. 1 in Nelson et al. [18], it is apparent that autopsy detected silicosis (including those with “occasional” or “few” palpable nodules) rose steadily from about 5 % of autopsies in 1984 to 15 % in 2000 to a period mean of about 24 % in 2000–2007 (crude proportions). Given that at least two thirds of the autopsy detectable silicosis may be subradiological in this population [23], the prevalence of pathological silicosis at autopsy would be expected to be considerably higher than that found radiologically. The absolute prevalence figures are thus not comparable. However, the trend over time between the autopsy series and that suggested by the current study is contradictory.

### Possible explanations for study discordance with autopsy data

We considered four possible explanations for a possible recent decline in silicosis prevalence in this one shaft, and flat trend overall compared to the 1984 study: radiological misclassification of silicosis, bias from selection into the study, declining silicosis risk due to falling exposure to respirable crystalline silica and an increasing healthy worker survivor effect.

#### Radiological misclassification

Some uncertainty was introduced into interpretation of study results by lack of inter-reader agreement. The kappa value was lower than that reported, for example, in the 2000 study [12]. Reader I has participated in previous studies and his readings have been validated against autopsy [21], while reader II has been validated against other expert readers (Churchyard 2004; Steen 1997; Girdler-Brown 2008) and was found in one study to have the best overall agreement among four experienced readers [16]. Additional file 1: Table S1b suggests that some of the discordance may have been due to reader I being more likely to record silicosis in the presence of signs of tuberculosis than reader II. While reader II was regarded as the “gold standard” in the inter-reader comparison, both readings have been presented.

#### Selection bias into the study

The overall participation rate among male employees in the Thibela TB study was 48.7 %, varying by shaft. Chest radiographs were not available from those who elected not to participate. However, a strong selection bias into the study by silicosis status or risk, and in particular avoidance of the study by those who knew they had silicosis, is unlikely for a number of reasons. The Thibela TB study was focused on tuberculosis rather than on silicosis. The chest radiograph was extracted from routine mine medical surveillance rather than being taken for research purposes. Most of the non-participation was among miners who did not attend the study centre to hear about the study. Finally, miners older than 44 years of age, with a much greater risk of silicosis, were slightly more likely to participate in the Thibela TB study than younger miners.

#### Declining respirable silica levels

It is possible that a real drop in silicosis incidence has taken place (or at least a shift from radiological to sub-radiological silicosis), and is related to decreased cumulative exposure to respirable crystalline silica dust. Such a decline in dust exposure would have had to have been effective from the early 1980s since the latency for radiological silicosis is around two decades. However, the evidence that is available is not consistent with a sustained decline in respirable crystalline silica dust concentrations over the period of concern. At the particular mine shaft where the 2000 study [12] was carried out, average particle counts as measured by konimeter rose between 1978 and 1984 from 174 to 258 ppcc, before falling to 212 ppcc in 1986 (informal occupational exposure limit: 200 ppcc) (te Water Naude J, Myers JM, Trends in dust levels at a gold mine near Orkney in the North West province, unpublished report, 2003). The same source showed that once gravimetric measurement dust concentrations were introduced, respirable dust concentrations rose over the period 1992/3 to 1996 (from 0.5 to 0.66 mg/m^3^), with a decline in respirable free silica concentrations [from 0.13 to 0.09 mg/m^3^, occupational exposure limit: 0.1 mg/m^3^] owing to an apparent halving of the silica fraction of the dust from 26 to 12 % measured by infrared spectrometry.

#### Healthy worker survivor effect

After adjusting for potential confounding, having silicosis at baseline increased the risk of exiting the workforce by over 50 % over the follow up period and approximately 70 % among those with previous or active tuberculosis. Pressures for such selection out may be illness related or administrative. Illness related factors include respiratory impairment due to silicosis or tuberculosis, or departure during prolonged tuberculosis treatment, particularly for drug resistant tuberculosis. Administrative reasons derive from the Occupational Diseases in Mines and Works Act (ODMWA) [24], which prohibits miners with certified “second degree” occupational disease – in this case silicosis plus tuberculosis – from remaining in dusty work. These pressures are likely to explain why miners with combined disease at baseline showed no greater risk of exiting in the subsequent period than those with one disease alone. Miners with combined disease are less likely on administrative grounds than those with a single condition to have been present in the workforce for recruitment into the Thibela TB study, while those remaining are likely to be at the “healthier” end of the combined disease spectrum.

To explain a falling prevalence of silicosis in this one shaft during the period 2000 to 2008, the above processes would have to have accelerated or changed in recent years. There are some reasons why this may be so.

In line with the generalised epidemic in South Africa, HIV infection rates among miners increased rapidly in the 1990s. Although published representative HIV data are scarce, an HIV seroprevalence of 27 % was recorded among gold miners attending routine examinations in 2000–2001 [6], compared to 13 % recorded in a high risk subpopulation of miners in 1995 [25]. The close association of HIV with tuberculosis and its multiplicative interaction with silicosis [6] are likely to have greatly increased the risk of tuberculosis, its recurrence [26] and associated comorbidity [27], and with these, the likelihood of employment loss due to ill-health over this decade.

Gold mining employment in South Africa is shrinking. Gold mining employment fell from approximately 475 000 in 1990 to 200 000 in 2002 to 160 000 in 2009 [28, 29]. This is accompanied by high turnover. For example, approximately 793 000 South African workers were recruited into the mining industry (all commodities, i.e. including gold, platinum and coal) in the period 2003–2013, while approximately 508 000 had their employment terminated [30]. There has also been a large shift in the distribution from gold to platinum mining, with employment in the platinum mining industry exceeding that of the gold mining industry in 2006 [29].

In terms of ODMWA mentioned above, miners with either silicosis or tuberculosis on its own (“first degree” disease) are supposed to be clinically assessed and reported for compensation claim purposes, but are permitted to remain in dusty work [22]. However, with the decline of the gold sector, pressures for such miners to leave gold mining employment may be increasing.

## Conclusions

In summary, the results of this large multi-company survey of working miners in the South African gold mining industry conducted between 2004 and 2009 suggest that the prevalence of radiological silicosis among black miners is similar to what it was 20–25 years previously. The findings of a recent decline at one shaft and a flat trend overall in silicosis prevalence among active black miners are discordant with the rising autopsy silicosis trend over this period. Taken together with a strong healthy worker survivor effect, these findings suggest that a transfer of the burden of silicosis and related tuberculosis to labour sending areas or into other mining sectors continues and is probably increasing. The study is notable in being able to demonstrate empirically the healthy worker survivor effect.

A recent study by the World Bank estimated there are about 1.5 to 2 million ex-mine workers spread over 4 southern African countries – namely, South Africa, Lesotho, Mozambique and Swaziland [30]. The study further noted that the South African mining population has the highest incidence of TB of any working population in the world, and that prolonged silica dust exposure and silicosis are major risk factors for tuberculosis, along with HIV. Consequently, Ministries of Health in the affected countries, the Global Fund, the World Bank, and the Stop TB Partnership among others are collaborating on a proposed public health initiative to control tuberculosis and its risk factors across the subcontinent [30]. A primary component of such a programme will be to identify miners and ex-miners with tuberculosis and silicosis [8]. The findings of the study reported here should provide useful information for this subcontinental initiative.

In addition, the findings have two other important implications. First, for the mining industry, government, SADC and other stakeholders to be able to determine whether progress is being made in the control of factors driving silicosis and tuberculosis in the region, better surveillance of silicosis is required. Elements of improved surveillance include harmonised methods for diagnosing and reporting silicosis, improved access to diagnosis by ex-miners, an information system capable of capturing and integrating data from these activities and the epidemiological capacity to interpret and publish the resulting information. Contrary to its legal mandate under the ODMWA [22], the Medical Bureau for Occupational Diseases, the agency responsible for certifying silicosis and related tuberculosis, has not produced an annual report since 1999 [31]. Such surveillance activities would have to be undertaken across all the labour sending Southern African countries – a formidable task.

Second, the large population of former gold miners is likely to carry an elevated lifelong risk of tuberculosis from previous exposure to respirable crystalline silica dust, even in the absence of radiological silicosis [3–5], as well as the risk of new or progressive silicosis after leaving gold mining employment [32]. Accordingly, studies of the long-term trajectory of silicosis and tuberculosis need to include former and not only current gold miners to further understand disease onset following the end of employment, progression of disease, and impact on lung function and survival.

## Ethics approval

The Thibela TB study was approved by the University of Kwazulu Natal Biomedical Research Ethics Committee and the London School of Hygiene and Tropical Medicine Ethics Committee. Approval for the silicosis analysis was subsequently obtained from the University of Kwazulu Natal Biomedical Research Ethics Committee and the Research Ethics Committee of the Faculty of Health Sciences, University of Cape Town.

## Additional file

Additional file 1: Table S1. Inter-reader agreement on silicosis. a: Inter-reader agreement on silicosis: reader I versus reader II. b: Inter-reader agreement on silicosis: reader I vs. reader II, stratified by whether reader I read signs of tuberculosis as present or absent. (DOCX 18 kb)

## Footnotes

1 “Black miners” refers to the population, mainly migrants recruited from rural areas in South Africa and neighbouring countries, who make up the large majority of production workers on the mines and who have been and continue to be subject to similar working and living conditions and related disease risks. While disease risks overlap with those of “white miners”, particularly for silicosis, there are sufficient epidemiologically and socially important differences for the groups to be considered separately.
