# Supplementary material for: Trends in silicosis prevalence and the healthy worker effect among gold miners in South Africa: a prevalence study with follow up of employment status
Source: BMC Public Health. 2015 Dec 18;15:1258. doi: 10.1186/s12889-015-2566-8 (PMC4684919; doi:10.1186/s12889-015-2566-8)
Supplement: Additional file 1: Table S1. — Inter-reader agreement on silicosis. a: Inter-reader agreement on silicosis: reader I versus reader II. b: Inter-reader agreement on silicosis: reader I vs. reader II, stratified by whether reader I read signs of tuberculosis as present or absent. (DOCX 18 kb) [file 12889_2015_2566_MOESM1_ESM.docx]

**Supplemental Table 1: Inter-reader agreement on silicosis**

**Table 1a: Inter-reader agreement on silicosis: reader I versus reader II**

|  |  | | **Reader II** | | |  |
| --- | --- | --- | --- | --- | --- | --- |
|  |  | < 1/1* | | > 1/1 | Total |  |
| **Reader I** | < 1/1 | 365 | | 17 | 382 |  |
|  | >1/1 | 263 | | 196 | 459 |  |
|  | Total | 628 | | 213 | 841 |  |
| *International Labour Organisation (ILO) grade  Kappa =0.36 [95% confidence interval (CI) 0.31, 0.41]; agreement 67%. | | | | | | |

**Table 1b: Inter-reader agreement on silicosis: reader I vs. reader II, stratified by whether reader I read signs of tuberculosis as present or absent**

| **Signs of tuberculosis read as present by reader I** | | | | |  | **Signs of tuberculosis read as absent by reader I** | | | | |
| --- | --- | --- | --- | --- | --- | --- | --- | --- | --- | --- |
|  |  | **Reader II** | | |  |  |  | **Reader II** | | |
|  |  | < 1/1* | >1/1 | Total |  |  |  | < 1/1* | >1/1 | Total |
| **Reader I** | < 1/1 | 21 | 4 | 25 |  | **Reader I** | < 1/1 | 344 | 13 | 357 |
|  | >1/1 | 76 | 71 | 147 |  |  | >1/1 | 187 | 125 | 312 |
|  | Total | 97 | 75 | 172 |  |  | Total | 531 | 138 | 669 |
| *International Labour Organisation (ILO) grade  Kappa = 0.15 (95% CI 0.06, 0.24); agreement 53%. | | | | | Kappa = 0.38 (95% CI 0.32, 0.44); agreement 70%. | | | | | |
